# Reduction in Use of Risperidone for Dementia in Australia Following Changed Guidelines

**DOI:** 10.3390/pharmacy7030100

**Published:** 2019-07-22

**Authors:** Lisa M Kalisch Ellett, Anna K Moffat, Svetla Gadzhanova, Nicole L Pratt, Jemisha Apajee, Michael Woodward, Elizabeth E Roughead

**Affiliations:** 1 Quality Use of Medicines and Pharmacy Research Centre, School of Pharmacy and Medical Sciences, University of South Australia, GPO Box 2471, Adelaide SA 5001, Australia; 2 Austin Health, GPO Box 5444, Heidelberg West, Victoria 3081, Australia

**Keywords:** behavioural and psychological symptoms of dementia (BPSD), risperidone, antipsychotics, medication use restrictions, potentially inappropriate medicines, older people

## Abstract

**Background:** Risperidone is the only antipsychotic approved in Australia for the management of the behavioural and psychological symptoms of dementia (BPSD). In June 2015, the Australian Government Therapeutic Goods Administration (TGA) amended the indication to restrict use in BPSD to patients with Alzheimer’s dementia for a maximum twelve-week duration. We aimed to determine whether the rate and duration of risperidone use for BPSD decreased following the regulatory changes. **Methods:** we conducted a study using the Australian Government Department of Veterans’ Affairs administrative claims data and Pharmaceutical Benefits Scheme (PBS) 10% sample data. We included people aged 65 years or older and compared the rate and duration of risperidone use before and after the TGA labelling changes. **Results:** There was a sustained decrease in the trend of risperidone use for BPSD following the TGA labelling changes, with a monthly decrease of 1.7% in the aged care population, 0.5% in the community living population and 1.5% in the general older Australian population. Overall, in the 24 months post the TGA changes the reduction in the rate of use of risperidone ranged from 20% to 28% lower than compared to what the rate would have been without the TGA changes. The median duration of use of risperidone in aged-care residents decreased from 338 days in the year prior to the TGA labelling changes, to 240 days per person in the year after the changes. **Conclusion:** The TGA labelling changes were associated with a significant reduction in the rate of use of risperidone for BPSD in veterans living in both the aged care and community settings, and in the general older Australian population. The labelling changes were also associated with a reduced duration of risperidone use in aged care residents, although for most people the duration of use still exceeded the recommended 12-week maximum duration.

## 1. Introduction

It is estimated that there are currently more than 400,000 Australians living with dementia, [1] and over 90% of them will experience behavioural and psychological symptoms of dementia (BPSD) at some point in the course of their illness [2,3]. BPSD includes potentially severe, non-cognitive symptoms like apathy, depression, aggression, psychotic symptoms, sleep problems, wandering, calling out and agitation [4,5]. The frequency, severity and number of BPSD symptoms increases as dementia progresses into later stages [2,3]. Caregivers often find BPSD difficult to manage and BPSD is associated with earlier admission into residential aged care facilities (RACFs) [3,6]. More than half of residents in aged-care facilities in Australia have a diagnosis of dementia [7] and the prevalence of BPSD is higher in people with dementia who live in aged care than in people with dementia who live in the community [8]. 

Antipsychotic medicines are commonly used for the management of BPSD, despite evidence showing that they are no better than placebo at improving BPSD symptoms (measured using the Neuropsychiatric Inventory), and are associated with increased risk of serious adverse events including cerebrovascular adverse events, extrapyramidal side effects, and somnolence or sedation [9]. Individual studies have also shown an association between the use of antipsychotics and increased risk of other serious adverse events including hip fracture, pneumonia and death [10,11]. Australian clinical practice guidelines recommend that non-pharmacological strategies be considered as first line therapy for BPSD and that if pharmacological management is required, this should be in conjunction with non-pharmacological strategies, for the shortest time possible and regularly reviewed [12]. Previous research has shown that at least one in five residents in RACFs in Australia are prescribed regular antipsychotics [13,14] and 44% of residents dispensed anti-dementia medicines received antipsychotics concurrent to their anti-dementia medicines [5], indicating that use of antipsychotics to manage dementia is common in the aged care setting.

Risperidone is the only antipsychotic approved in Australia for the management of BPSD [15]. In June 2015, the Therapeutic Goods Administration (TGA) amended the indication for risperidone to restrict use in BPSD to patients with Alzheimer’s dementia for a maximum twelve-week duration [15]. Indications for BPSD in other dementias, including vascular dementia were removed. Prior to this change, there was no restriction on duration of use [15]. These changes occurred in response to increasing evidence about the harms of antipsychotics in patients with dementia, in particular with regards to stroke risk [15]. Information regarding the changes to the approved indications and duration of use for risperidone in dementia were publicised two months later in August 2015, in the TGA medicine safety update bulletin [15], the news media [16] and in targeted current affairs publications for prescribers [17,18]. A subsequent educational program was implemented in September 2016 [19]. The extent to which use of risperidone for BPSD reduced following publication of the TGA labelling changes has not been assessed. Therefore, we aimed to determine whether there was a reduction in the rate and duration of risperidone use for BPSD following the TGA labelling changes. 

## 2. Materials and Methods

We conducted a study using Australian Government Department of Veterans’ Affairs (DVA) administrative claims data and Pharmaceutical Benefits Scheme (PBS) 10% sample data, from 1 January 2012 until 31 August 2017. The DVA administrative claims data included approximately 200 000 people during this time period, [20] and contains details of all prescription medicines, medical and allied health services, aged care admissions and hospitalisations for which DVA pay a subsidy. The DVA data also includes a client file, which includes data on gender, date of birth, date of death and family status. Medicines are coded in the DVA dataset according to the World Health Organization (WHO) anatomical and therapeutic chemical (ATC) classification [21] and the Schedule of Pharmaceutical Benefits item codes [22]. The PBS 10% sample dataset contains claims for medicines dispensed on the Pharmaceutical Benefits Scheme to a random 10% sample of Australians [23]. Information relating to patient gender and year of birth or death are available in the PBS 10% sample dataset; however, information on whether the person lives in the community or aged care is not available. 

### 2.1. Assessment of Changes in the Rate of Use of Risperidone in Veteran Residents of Aged Care Facilities (DVA Data)

In each month of the study period, we identified a cohort of veterans who were alive, permanent residents in an aged care facility and aged 65 years or over in that month. We excluded people who were receiving respite care in an aged care facility. The monthly rate of use of risperidone from 2012 to 2017 was calculated as the proportion of people dispensed risperidone for dementia in that month within the overall study population for that month. Risperidone for dementia was identified by the PBS item codes “01842Y”, ”08787L”, “08789N”, “08788M”, ”08790P”, ”08791Q”, or “09293D” [22]. Supply of risperidone on these PBS item codes in people aged over 18 years is restricted to use for the management of BPSD in people with Alzheimer’s type dementia [22].

### 2.2. Comparison Cohorts: Assessment of Changes in the Rate of Use of Risperidone in Community Dwelling Older Veterans, and the General Australian Population

The prevalence of BPSD is higher in aged care residents with dementia than community dwelling people with dementia [8], so the effect of the TGA labelling changes on the rate of risperidone use may be different in the community dwelling population. Therefore, in each month of the study period, we used the DVA claims dataset to identify a comparison cohort of veterans who were alive, living in the community (i.e., not in permanent or respite aged care) and aged 65 years or over in that month. We calculated the monthly rate of use of risperidone for dementia within this community dwelling cohort using the same methods as we used for the aged care cohort.

The DVA treatment population has an older age distribution than the general Australian population aged 65 and over; therefore, the rate of use of risperidone in the general Australian population may differ to the veteran population. We therefore replicated the analysis, using data from the Pharmaceutical Benefits Scheme (PBS) 10% sample dataset. In each month of the study period, we identified people in the PBS 10% sample dataset who were dispensed risperidone for BPSD. Because exact date of birth is not available in the PBS 10% sample dataset, for each month of analysis we included anyone who was aged 65 or over in that year (based on the year of birth variable). The monthly population rate of use of risperidone for dementia from 2012 to 2017 was calculated as the proportion of people dispensed risperidone for dementia in that month (multiplied by 10 to account for the 10% sample) amongst the Australian population aged 65 and over based on estimates from Australian Demographic Statistics for the given period. 

### 2.3. Statistical Analysis of Changes in the Rate of Use of Risperidone

Interrupted time series modelling was used to determine the impact of the TGA labelling changes on the rate of use of risperidone for the aged care, community dwelling and general Australian population cohorts with one change point at August 2015, the date the TGA labelling changes were communicated to prescribers. We incorporated structural and autoregressive components into the model, and the analysis also controlled for the baseline trend, seasonality and any autocorrelation evident in the time series trend. Change-in-level terms were used to determine whether there were changes in the rate of use of risperidone immediately after the TGA labelling changes, and change-in-trend terms were used to determine whether there were more gradual changes in the trend of risperidone use after the TGA labelling changes. From this model, predicted rates were generated and the average month-to-month change (%) in the trends was calculated as the ratio of the model-estimated values in each month to the values from the previous month. The relative change in the use of risperidone associated with the TGA changes was expressed as a percentage increase or decrease from the predicted use without the TGA labelling changes.

### 2.4. Assessment of Changes in the Duration of Use of Risperidone in the DVA Aged Care Cohort

To assess whether duration of risperidone use had decreased following the TGA labelling changes, we assessed duration of use amongst veterans in the aged care cohort before and after the labelling changes. We limited this part of the analysis to the aged care cohort, because this is the population where risperidone use was most prevalent. This is also the population where BPSD is most prevalent [8] and so misclassification of use for other indications (e.g., psychosis) where long term duration of risperidone use is appropriate would be less likely. We split the cohort in to two time periods, aged care residents who were dispensed risperidone between 1 August 2012 to 31 July 2013 (pre TGA labelling change) and aged care residents who were dispensed risperidone between 1 August 2015 and 31 July 2016 (post TGA labelling change). We calculated their duration of use of risperidone for BPSD by summing the number of risperidone tablets or volume of syrup dispensed over the year and dividing by the usual number of daily doses for BPSD [24]. Duration of use was capped at 365 days. We excluded risperidone injection, because this is only indicated for the management of schizophrenia or bipolar disorder; not BPSD. We used the Wilcoxon test to determine whether the median duration of risperidone use decreased in the post-change period compared to the pre-change period.

This research was approved by the Australian Government Department of Veterans’ Affairs (DVA) Human Research Ethics Committee, the University of South Australia Human Research Ethics Committee and the Australian Government Department of Human Services External Request Evaluation Committee. All analyses were conducted using SAS, V9.3 SP4 (SAS institute, Cary, North Carolina, USA).

## 3. Results

### 3.1. Changes in the Rate of Use of Risperidone

**Figure 1:**
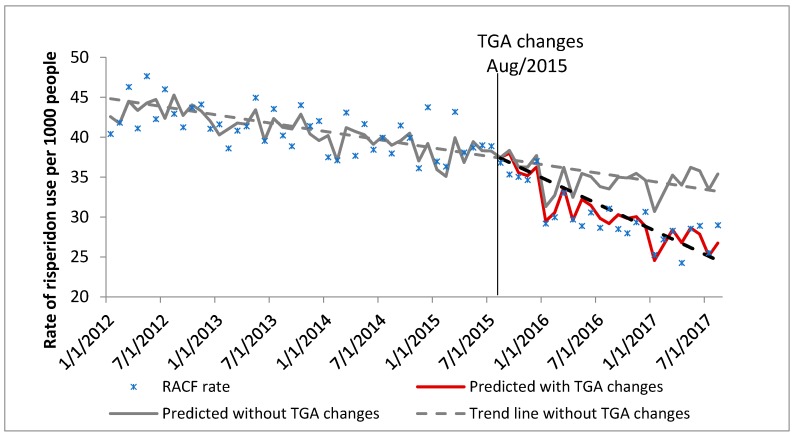
Aged care cohort: Rate of use of risperidone for behavioural and psychological symptoms of dementia (BPSD) in people aged 65 years and over, living in aged care (DVA data).

**Figure 2:**
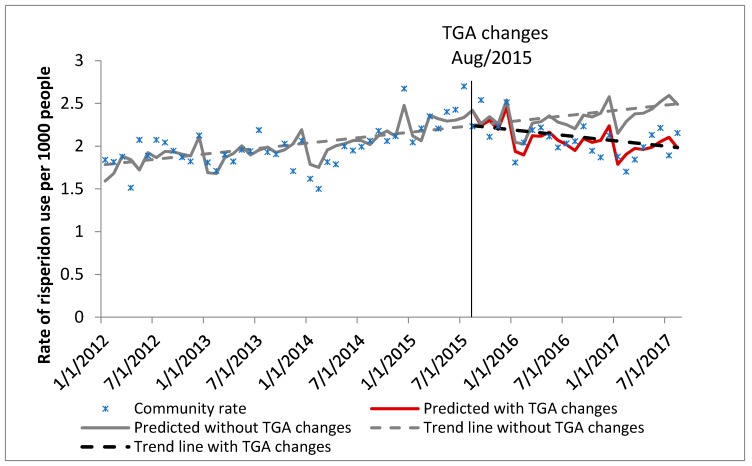
Community dwelling cohort: Rate of use of risperidone for BPSD in people aged 65 years and over, living in the community (DVA data).

**Figure 3:**
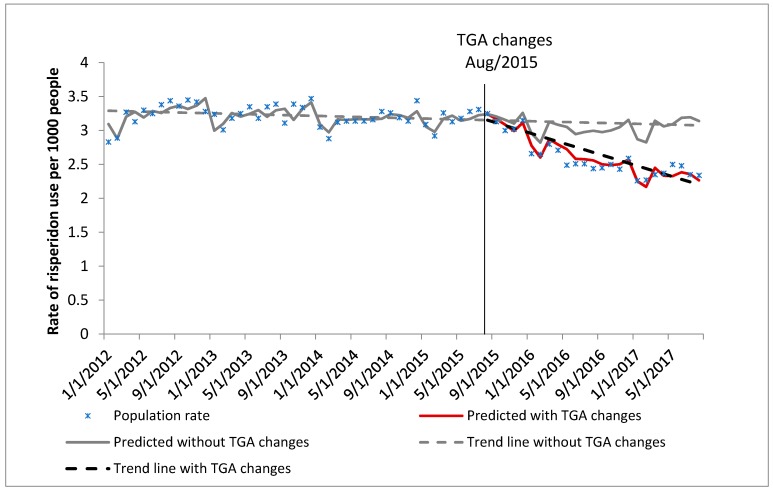
General Australian cohort (PBS 10% sample): Rate of use of risperidone for BPSD in people aged 65 years and over.

In January 2012 there were 28,949 veterans aged 65 years or over who were resident in aged care, with a median age of 86 years. By August 2017, this had decreased to 19,350 people with a median age of 92 years (Table 1). Characteristics of the DVA community dwelling cohort and the general Australian population cohort (PBS 10% sample data) are also shown in Table 1. The trends in the rate of use of risperidone for BPSD are shown in Figure 1 (DVA aged care cohort), Figure 2 (DVA community dwelling cohort) and Figure 3 (PBS general Australian population cohort).

DVA aged care cohort (Figure 1): prior to the TGA labelling changes, the rate of use of risperidone in the aged care cohort was approximately 45 people per 1000 veterans, and this rate was decreasing by 0.49% each month (compared with the previous month). Following the TGA labelling changes the monthly use was decreasing significantly by 1.74%. There was 26.07% relative decrease in the rate of use 24 months post the TGA changes (August 2017) compared to what the rate would have been at 24 months post changes had the labelling changes not occurred (Table 2). 

DVA community dwelling cohort (Figure 2): Prior to the TGA labelling changes, the rate of risperidone use for BPSD in the community dwelling cohort was approximately two people per 1000 veterans, and this rate was increasing by 0.45% each month. Post TGA changes the rate was decreasing significantly by 0.5% from month to month. There was 20.44% relative decrease in the rate 24 months post TGA labelling changes compared to the predicted rate without the TGA changes (Table 2).

General Australian population (PBS 10% sample cohort, Figure 3): Prior to the TGA labelling changes, the rate of use of risperidone for BPSD in the general Australian population aged 65 years and over was approximately 3.5 people per 1000 population, and this rate was decreasing by 0.1% each month. Following the TGA labelling changes, the rate of use of risperidone was decreasing significantly by 1.49% from month to month. There was 28.42% relative decrease in the rate 24 months post TGA changes compared to the rate if the changes have not occurred (Table 2).

### 3.2. Changes in the Duration of Use of Risperidone in DVA Aged Care Cohort

**Figure 4:**
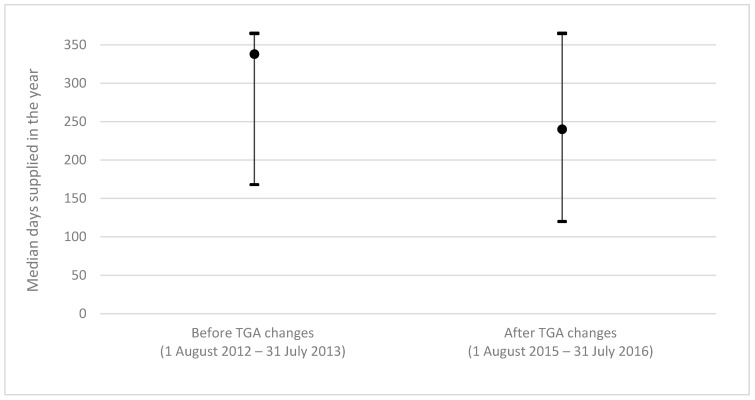
Median duration of risperidone use in aged care residents in one-year periods, before and after TGA labelling changes (DVA data). **Figure legend:** Median duration of risperidone use decreased from 338 days per person in the year before the TGA labelling changes to 240 days per person in the year after the TGA labelling changes (*p* < 0.0001).

In 2012/13, before the TGA labelling changes, there were 17,785 veterans living in aged care who were dispensed risperidone. They had a median age of 89 years and 12,785 (71.9%) were female. In 2015/16, after the TGA labelling changes, there were 14,004 veterans living in aged care who were dispensed risperidone and they had a median age of 91 years (IQR 88-93). Seventy six percent of people dispensed risperidone in aged care were women (*n* = 10,684). The median duration of risperidone use significantly decreased from 338 days (IQR 168-365) in 2012/13 to 240 days (IQR 120-365) in 2015/16 (*p* < 0.0001) after the TGA labelling changes (Figure 4). 

## 4. Discussion

Our study shows that following the TGA labelling changes in 2015, the rate of use of risperidone for BPSD decreased. The largest monthly decrease in the rate of use of risperidone was evident in the aged care population, where the decrease was by 1.7% each month following the labelling changes. This decrease was three times higher than the decrease in the rate prior to the TGA changes. In the DVA community dwelling cohort the rate of use of risperidone for BPSD was increasing each month prior to the TGA labelling changes; this trend was reversed after the TGA labelling changes, with a monthly decrease of 0.5%. In the general Australian population cohort (PBS 10% sample), there was a monthly decrease of 1.5% post TGA changes compared to a decrease of 0.1% only without to the changes. The relative effect sizes of the TGA labelling changes in August 2017 (24 months post the changes) ranged from a 20% to 28% reduction in the rate of use of risperidone compared to what the rate would have been without the TGA changes. 

The clinical significance of these changes in risperidone use are likely to be substantial. In the aged care cohort, which had the highest baseline rate of risperidone use, the decreased rate of use of risperidone was equivalent to 2,244 fewer patient months of treatment in the 24 months after the TGA labelling changes, or 94 fewer people using risperidone for each month in the two years after the labelling changes. A previous study conducted in the DVA population found that for every 12 weeks of atypical antipsychotic use in older people there was one additional hip fracture per 40 people treated and one additional case of pneumonia per 13 people treated, that would not have occurred had the antipsychotic not been used [10]. Applying these risk estimates to the aged care population in our study suggests that 18 hip fractures and 57 cases of pneumonia were avoided amongst veterans in aged care as a result of the decreased use of risperidone following the TGA labelling changes.

Our study is the first to assess changes in the rate of use of risperidone in Australia following the TGA regulatory changes in 2015. Several international studies have assessed the impact of regulatory safety warnings on the use of antipsychotics for dementia using interrupted time series analysis, with varying results. A French study assessed the rate of use of all antipsychotics in people with dementia and found that, although the rate of antipsychotic use in this population significantly decreased between 2003 and 2011, the rate of decrease did not significantly change when regulatory warnings were issued by the French medicines regulator in 2004 and 2008 [25]. In Scotland, regulatory warnings relating to the use of antipsychotics in people with dementia were issued in 2004 and 2009 [26]. The 2004 communication was associated with significant decreases in both the level and post-intervention trend of antipsychotic prescribing, while the 2009 intervention was associated with a significant decrease in the post-intervention trend of antipsychotic prescribing [26]. Another study compared the effect of regulatory warnings on antipsychotic use in the UK and Italy and found that there was a decrease in the use of antipsychotics in both countries following a 2004 regulatory warning, but the trend was increasing again one year later [27]. Following a subsequent regulatory warning in 2009 there was another decrease in the prescribing trend in the UK but an increasing trend in Italy [27]. In Canada, a regulatory warning in 2002 was associated with a significant decrease in both the level and trend of risperidone use in people with dementia; however, the overall rate of risperidone use continued to increase in this study population after the regulatory warning [28].

Results of these studies are not directly comparable to our study due to differences in the medicines available for use in BPSD in each setting and the patient populations included in the analyses. However, the variable results do highlight the difficulty in changing healthcare behaviour to improve medicines use. Strategies to improve the use of medicines need to be multifactorial in order to be successful and changing regulations around medicines use without providing clinicians with information about alternative ways to manage the condition is likely to have limited effectiveness in improving medicines use [29]. Although the risperidone labelling changes were publicised in TGA bulletins [15] and the news media [16], none of these publications provided guidance for prescribers on appropriate, evidence based alternative management strategies for BPSD. A subsequent educational intervention directed towards veterans and the GPs caring for them was implemented in September 2016, and this intervention provided prescribers with advice on non-pharmacological management strategies for BPSD [19]. This intervention is likely to have supported the sustained decreasing trend in risperidone use in both of the DVA cohorts following the intervention. 

The adverse events associated with risperidone use are serious and there is limited evidence for the efficacy of risperidone in managing BPSD symptoms, [30,31,32] so the decision to use risperidone for the management of BPSD should only be made after other management strategies have been trialled and have failed. However, managing challenging behaviours in people with dementia can be difficult and a recent systematic review highlighted the competing pressures that influence the decision to use antipsychotics for BPSD in nursing home residents [33]. These included staffing pressures and the difficulty in finding the time to provide patient centred care (which can reduce the need for antipsychotics), lack of access to specialists like psychiatrists to assist in the management of BPSD, and the feeling that in many cases doctors have no other option but to prescribe antipsychotics to manage severe behavioural problems in people with BPSD [33]. There are no PBS-subsidised alternatives to risperidone for BPSD and prior studies have indicated that non-pharmacological management of BPSD is limited by staffing resources and skills [33]. In the face of these difficulties, the reduction in the use of risperidone in both aged care residents and community dwelling people with dementia is encouraging. 

Although the duration of use of risperidone decreased in the aged care population following the TGA labelling changes, from a median of 338 days per person prior to the changes to 240 days after, this was still in excess of the new 12-week (84 days) duration recommended by the TGA. Sustaining practice change in healthcare is difficult, and repetition of messages has been shown to improve the duration of healthcare improvements following healthcare interventions [34]. Future health improvement interventions will support a sustained reduction in the rate of use of risperidone for BPSD. These interventions should also focus on the importance of reducing the duration of use of risperidone in people who need to use it for BPSD.

There are several limitations to our study. Diagnoses are not recorded in the DVA medicine claims data or PBS 10% sample dataset, so we were unable to determine whether risperidone use was for patients with Alzheimer’s or other dementia types. The odds of vascular adverse events with risperidone are markedly raised in those with vascular or mixed dementia (odds ratio 5.26, 95% confidence interval 1.18–48.11) [15], so assessing the appropriateness of risperidone use by dementia type is an important focus for future research. We were unable to reliably identify people with and without dementia in the medicines claims data sets used for this study, so we were unable to stratify our results by people with and without a dementia diagnosis. We identified use of risperidone for BPSD by PBS item codes where use is restricted to the management of BPSD. Although the supply of risperidone on these PBS item codes should be limited to BPSD, the validity of this assumption has not been assessed and it is possible that some misclassification of risperidone use for other indications occurred.

## 5. Conclusions

The TGA labelling changes were associated with a significant reduction in the rate of use of risperidone for BPSD in veterans living in both the aged care and community settings, and in the general older Australian population. The labelling changes were also associated with a significant reduction in the duration of risperidone use in aged care residents, although for most people the duration of use still exceeded the recommended 12-week maximum duration. 

## Figures and Tables

**Table 1:** Demographics of the cohorts at study start, the date of the Therapeutic Goods Administration (TGA) labelling changes and at study end.

		Veterans Aged 65 and over Living in Aged Care *	Veterans Aged 65 and over Living in the Community *	General Australian Cohort Aged 65 and over #
Study start date: 1 January 2012	Total N	28,949	160,282	411,546
	N Men (%)	10,734 (37.1%)	85,089 (53.1%)	195,305 (47.5%)
	Median age (IQR)	89 (86–91)	85 (77–88)	74 (69–82)
Date of intervention: 1 August 2015	Total N	23,141	136,196	490,757
	N Men (%)	7283 (31.5%)	77,759 (57.1%)	235,869 (48.1%)
	Median age (IQR)	91 (88–93)	83 (70–90)	75 (69–83)
Study end date: 31 August 2017	Total N	19,350	122,072	544,858
	N Men (%)	5549 (28.7%)	72,533 (59.4%)	263,376 (48.3%)
	Median age (IQR)	92 (89–94)	80 (70–90)	75 (69–83)

* Department of Veterans’ Affairs (DVA) administrative claims data set; # PBS 10% sample dataset.

**Table 2:** Results from the interrupted time series models.

	Month-to-Month Change (%) in the Trends		Relative Effect (95% CI) at 24 Months (August 2017) Post the TGA Changes
	Trend without TGA Changes	Trend with TGA Changes	Trend with Versus Trend without TGA Changes
Veterans aged 65 and over living in aged care *	−0.49%	−1.74%	−26.07% (−10.89% to −41.26%)
Veterans aged 65 and over living in the community *	0.45%	−0.50%	−20.44% (−7.98% to −32.90%)
General Australian cohort aged 65 and over #	−0.10%	−1.49%	−28.42% (−11.55% to −45.30%)

* DVA administrative claims data set; # PBS 10% sample dataset.
